# Knowledge Domain and Emerging Trends in Ferroptosis Research: A Bibliometric and Knowledge-Map Analysis

**DOI:** 10.3389/fonc.2021.686726

**Published:** 2021-06-03

**Authors:** Jie Zhang, Luxia Song, Liyan Xu, Yixuan Fan, Tong Wang, Wende Tian, Jianqing Ju, Hao Xu

**Affiliations:** ^1^ Graduate School, Beijing University of Chinese Medicine, Beijing, China; ^2^ National Clinical Research Center for Chinese Medicine Cardiology, Xiyuan Hospital, China Academy of Chinese Medical Sciences, Beijing, China; ^3^ Department of Computer Science, Emory University, Atlanta, GA, United States; ^4^ Graduate School, China Academy of Chinese Medical Sciences, Beijing, China

**Keywords:** ferroptosis, bibliometric, knowledge-map, CiteSpace, VOSviewer

## Abstract

**Objectives:**

To identify the cooperation and impact of authors, countries, institutions, and journals, evaluate the knowledge base, find the hotspot trends, and detect the emerging topics regarding ferroptosis research.

**Methods:**

The articles and reviews related to ferroptosis were obtained from the Web of Science Core Collection on November 1, 2020. Two scientometric software (CiteSpace 5.7 and VOSviewer 1.6.15) were used to perform bibliometric and knowledge-map analysis.

**Results:**

A total of 1,267 papers were included, in 466 academic journals by 6,867 authors in 438 institutions from 61 countries/regions. The ferroptosis-related publications were increasing rapidly. *Cell Death & Disease* published the most papers on ferroptosis, while *Cell* was the top co-cited journal, publication journals and co-cited journals were major in the molecular and biology fields. The United States and China were the most productive countries; meanwhile, the University of Pittsburgh, Columbia University and Guangzhou Medical University were the most active institutions. Brent R Stockwell published the most papers, while Scott J Dixon had the most co-citations; simultaneously, active cooperation existed in ferroptosis researchers. Ten references on reviews, mechanisms, and diseases were regarded as the knowledge base. Five main aspects of ferroptosis research included regulation mechanisms, nervous system injury, cancer, relationships with other types of cell death, and lipid peroxidation. The latest hotspots were nanoparticle, cancer therapy, iron metabolism, and in-depth mechanism. Notably Nrf2 might have turning significance. The emerging topics on ferroptosis research were the further molecular mechanism of ferroptosis and the wider application of ferroptosis-related disease with advanced technology.

**Conclusion:**

This study performed a full overview of the ferroptosis research using bibliometric and visual methods. The information would provide helpful references for scholars focusing on ferroptosis.

## Introduction

Ferroptosis is an iron-dependent regulated necrosis caused by unrestricted lipid peroxidation and subsequent membrane damage (1). Scott J Dixon (2) proposed the term ferroptosis in 2012 to describe a type of cell death induced by the small molecule erastin, which could lead to glutathione (GSH) depletion and glutathione peroxidase 4 (GPX4) inactivation (1, 3). In addition to the necrotic morphological changes, ferroptotic cell death usually shows mitochondrial abnormalities, such as increased membrane density, reduced or absent crista, condensation or swelling, and rupture of the outer membrane (2, 4–7). Existing evidence proves that ferroptosis plays an important role in the development of many diseases (7, 8), such as cancer, neurodegenerative diseases, ischemia/reperfusion injury, acute kidney injury, atherosclerosis, chronic obstructive pulmonary disease, and immune system diseases. Therefore, as an evolutionary program offers various druggable nodes, ferroptosis is supposed to be an emerging way to cure many kinds of diseases (9–14). Especially as a therapeutic model in cancer treatment and prevention of ischemic organ damage, ferroptosis has been convincingly established (15).

According to its great potential, ferroptosis has gained scholars’ keen interest in better understanding the process of ferroptosis with a rapidly increasing number of publications (16). Many scholars have reviewed ferroptosis research from various aspects. For instance, Daolin Tang etc. (7) summarized the progress of ferroptosis research mainly from molecular mechanisms, including hallmarks, regulation, oxidant system, antioxidant system, membrane repair, degradation systems, transcription factors and cofactors, epigenetic regulation, assays, and the implications in disease. Xuejun Jiang etc. (17) overviewed ferroptosis from the mechanisms, pathways, biological functions in tumor suppression and immune surveillance, and the implications in cancer and ischemic damage. Marcus Conrad etc. (12) outlined ferroptosis mainly from its effects on as-yet- incurable disease, including ischemia-reperfusion injury, organ failure, neurodegenerative disease and therapy-resistant cancer. However, there is no comprehensive and objective report on the publication trends, influential authors or institutions and their cooperation, knowledge base, hotspots evolution, or the emerging topics in ferroptosis research to our knowledge.

Nowadays, there are kinds of approaches to systemically review a research field, of which bibliometrics is one of the most popular methods (18). Bibliometrics can not only qualitatively and quantitatively analyze the contribution and cooperation of authors, institutions, countries, and journals, but also evaluate the development and emerging trends in scientific research (18–23). That which other methods, such as traditional review, meta-analysis, or experiment research, cannot perform. According to the strengths, it is becoming increasingly important in evaluating research trends and formulating guidelines (24). Therefore, bibliometrics is suitable for evaluating and overviewing ferroptosis research.

This study aimed to use two commonly used bibliometric tools, CiteSpace and VOSviewer, to objectively describe the knowledge domain and emerging trends of ferroptosis research from three aspects as follows. (1) We designed to quantify and identify the general information in ferroptosis research, such as the individual impact and the cooperation information, by analyzing annual publications, journals, co-cited journals, countries/regions, institutions, authors and co-cited authors. (2) We planned to find and analyze the most co-cited papers by co-cited reference analysis to evaluate the knowledge base of ferroptosis. (3) Most importantly, finding the knowledge structure and hotspots evolution, and detecting the emerging topics of ferroptosis by keywords analysis and co-cited reference burst analysis. Overall, these three aspects cover the status quo and trends of ferroptosis research.

## Materials and Methods

### Data Collection

The Web of Science Core Collection (WoSCC) database is commonly used in bibliometric analysis (19, 22, 25–27). We also chose it because it can provide comprehensive information bibliometric software needs and is regarded as the most influential database (28).

Data were retrieved from the WoSCC database on November 1, 2020. We searched “ferroptosis” and “ferroptotic” as the term and set the timespan from the inception of Web of Science (WoS) to November 1, 2020. The language was restricted to English, and the article type was limited to Article or Review. Search results were downloaded with the record content of “Full Record and Cited References” and the file format of “Plain Text”. Then, we renamed the files for further analysis because CiteSpace can only recognize files named “download *.txt”.

### Data Analysis and Visualization

At present, the commonly used bibliometric software includes VOSViewer, CiteSpace, SCI2, NetDraw, and HistCite (29). There is no consensus on which bibliographic method is the best (30). Considering their characteristics and advantages, this research used both VOSviewer and CiteSpace (22, 25, 29, 31).

VOSviewer, developed by Leiden University, is a software that does well in creating, visualizing, and exploring maps based on network data (32, 33). We used VOSviewer 1.6.15 to identify productive journals, co-cited journals, authors, co-cited authors, as well as the related knowledge-maps based on bibliographic data. In addition, we created the keywords co-occurrence and cluster map based on text data. Terms were obtained from titles and abstracts fields using natural language processing algorithms and complemented with a VOSviewer corpus file (33). Firstly, we cleaned the data, such as merged “van raan, a” and “vanraan, a” in author analysis, unified “glutathione” and “gsh” as “gsh,” and deleted meaningless terms such as “focus” and “year” in term analysis (34). Secondly, we used fractional counting as the counting method and set the maximum number of authors per document as 25 (35). The difference between full counting and fractional counting is the strength of the links (33). The fractional counting method calculated the link strength by splitting papers according to the weight (18, 30, 33). For example, if three authors co-author a paper, each of their link strength will be counted as 1/3 in fractional counting, while it will be counted as one in full counting. It can be identified that fractional counting performs more reasonable in author analysis (36), and after comparing two methods in other sections, the data in our study showed more reasonably and clearly by the fractional counting method. Besides, in term analysis, each term was calculated a relevance score, which represented that terms with a high relevance score tend to represent specific topics, while terms with a low relevance score tend not to be representative of any specific topic (33). Therefore, we selected the terms that not only appeared more than ten times but also at the top 60% relevance score to analyze. Other thresholds (T) of items were set based on different situations (19), which were marked in corresponding tables and figures.

CiteSpace, developed by Prof. Chaomei Chen, is a bibliometric and visual analysis tool good at exploring cooperation, key points, internal structure, potential trends and dynamics in a certain field (37). Therefore, we used CiteSpace 5.7 to analyze and visualize the co-occurrence of countries/regions and institutions, dual-map of journals, trends of high-frequency keywords, co-cited references, and citation bursts for references. We cleaned the data before analyzing; for instance, in countries/regions analysis, publications from Taiwan were reclassified to China (35), and those from England, Scotland, Northern Ireland, and Wales were assigned to the United Kingdom (36). Similarly, we merged the synonyms such as “GPX4” and “glutathione peroxidase 4” in keyword evolution analysis. The CiteSpace settings were as follows: time span (2012–2020), years per slice (1), pruning (Minimum Spanning Tree and Pruning Sliced Networks), selection criteria (Top N=50), and others followed the default.

We used Microsoft Office Excel 2019 to manage the database and analyze the annual publications.

Besides, we obtained the 2019 impact factor (IF) and JCR division of journals from the Web of Science InCites Journal Citation Reports on November 15, 2020.

## Results

### Annual Growth Trend

**Figure 1:**
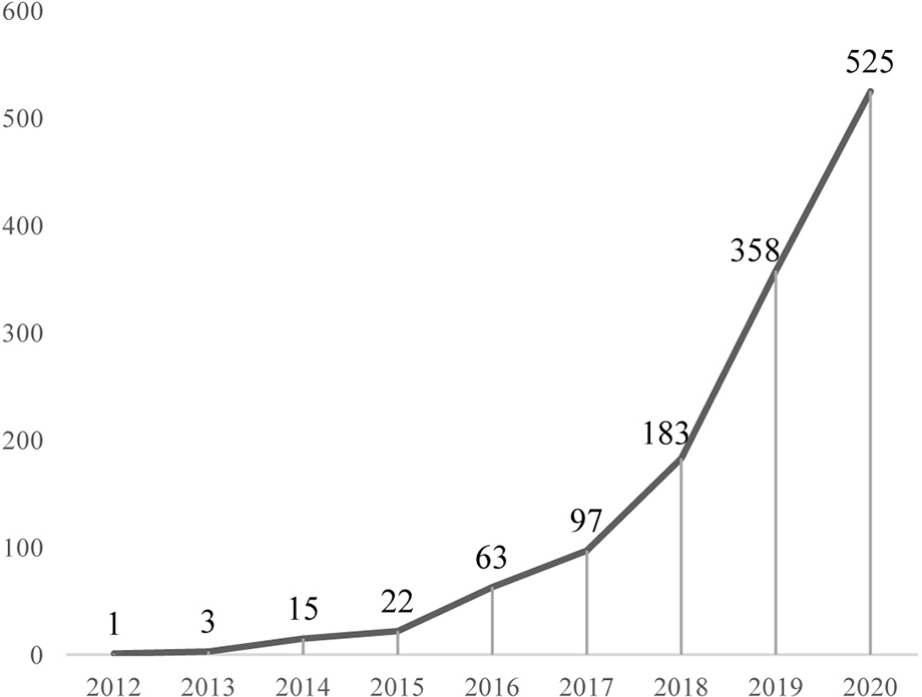
Annual output of ferroptosis research.

According to the data collection strategy, we collected 1,268 papers but these contained one duplicate. Finally, a total of 1,267 eligible papers were included (Annexes 1), published between 2012 and 2020. As we can see from **Figure 1**, ferroptosis-related references showed an annual upward tendency. Significantly, the yearly output is almost twice as much as the previous year in the last three years (2018 to present).

### Journals and Co-Cited Journals

We used VOSviewer to conduct co-citation and co-cited journal analysis, finding the most active and most influential journals in the ferroptosis field. The results showed that the 1,267 references were published in 466 academic journals. *Cell Death* & *Disease* published the most papers (41, 3.21%), followed by *Biochemical and Biophysical Research Communications*, *Free Radical Biology and Medicine*, *Redox Biology*, and *Cell Death and Differentiation* (**Table 1**). Among the top10 journals, seven were at the Q1 JCR division, and six had an Impact Factor (IF) of more than five (**Table 1**).

**Table 1:** The top 10 journals of ferroptosis research.

Rank	Journal	N (%)	IF(2019)^#^	JCR division	Country
1	Cell Death & Disease	41 (3.21%)	6.304	Q1	UK
2	Biochemical and Biophysical Research Communications	36 (2.82%)	2.985	Q2/Q3	USA
3	Free Radical Biology and Medicine	31 (2.43%)	6.170	Q1	USA
4	Redox Biology	27 (2.12%)	9.986	Q1	Netherlands
5	Cell Death and Differentiation	21 (1.65%)	10.717	Q1	UK
6	Oxidative Medicine and Cellular Longevity	20 (1.57%)	5.076	Q2	USA
7	International Journal of Molecular Sciences	20 (1.57%)	4.556	Q1/Q2	Switzerland
8	Scientific Reports	16 (1.25%)	3.998	Q1	UK
9	Frontiers in Neuroscience	15 (1.18%)	3.707	Q2	Switzerland
10	Cell Chemical Biology	15 (1.18%)	7.739	Q1	USA

^#^IF: Impact Factor.

Among 4,781 co-cited journals, eleven journals had citations over 1,000. As we can see from **Table 2**, *Cell* had the most co-citations (3,926, 4.21%), followed by *Nature*, *Journal of Biological Chemistry*, and *Proceedings of the National Academy of Sciences of the United States of America (PNAS)*. Among the top 10 co-cited journals, eight were at the Q1 JCR division with an Impact Factor (IF) of more than six, seven were from the United States.

**Table 2:** The top 10 co-cited journals of ferroptosis research.

Rank	Co-cited Journal	N (%)	IF(2019)^#^	JCR division	Country
1	Cell	3,926 (4.21%)	38.637	Q1	USA
2	Nature	2,912 (3.12%)	42.779	Q1	Germany
3	Journal of Biological Chemistry	2,661 (2.85%)	4.238	Q2	USA
4	Proceedings of The National Academy of Sciences of the United States of America	2,475 (2.65%)	9.412	Q1	USA
5	Cell Death and Differentiation	1,766 (1.89%)	10.717	Q1	UK
6	Free Radical Biology and Medicine	1,758 (1.88%)	6.170	Q1	USA
7	Nature Chemical Biology	1,270 (1.36%)	12.587	Q1	Germany
8	Plos One	1,152 (1.23%)	2.74	Q2	USA
9	Cancer Research	1,066 (1.14%)	9.727	Q1	USA
10	Science	1,061 (1.14%)	41.846	Q1	USA

^#^IF, impact factor.

**Figure 2:**
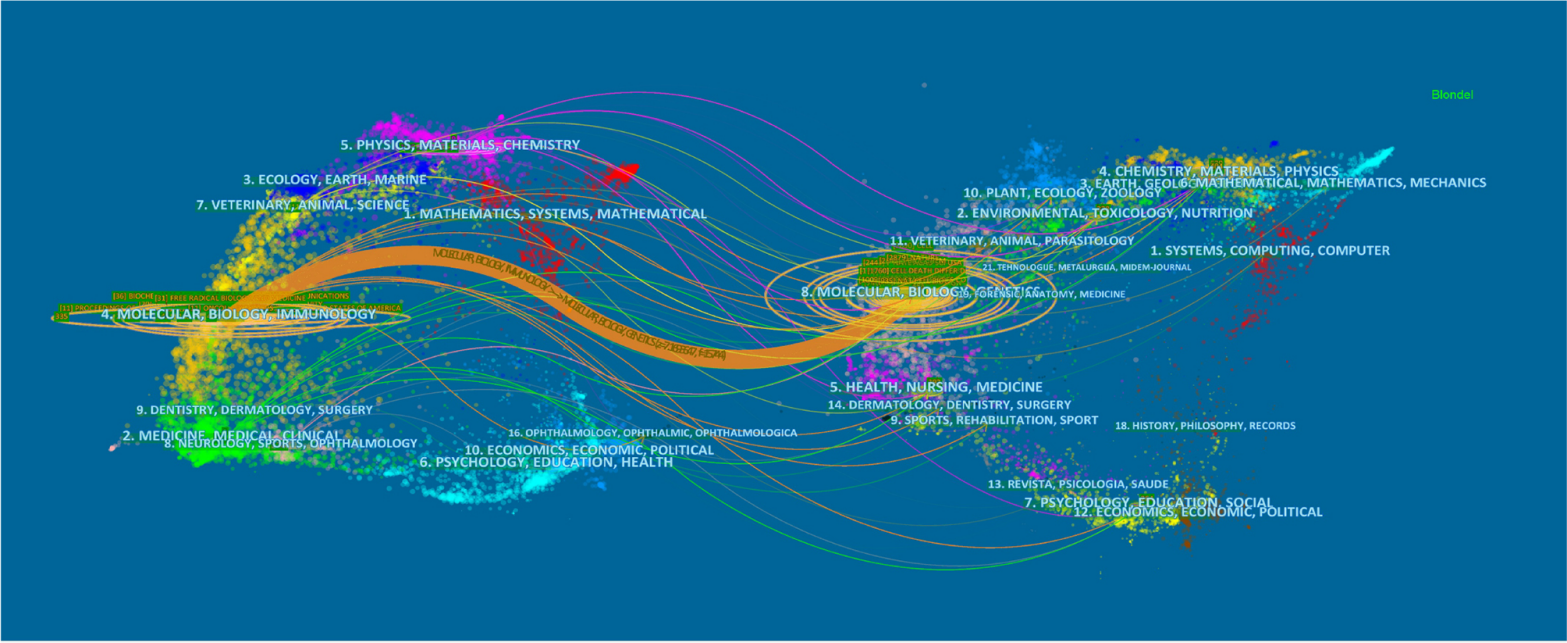
The dual-map overlay of journals related to ferroptosis research. Notes: The citing journals were at left, the cited journals were on the right, and the colored path represents citation relationship.

The dual-map overlay of journals stands for the topic distribution of academic journals (38) (**Figure 2**). The citing journals were located on the left while the cited journals were on the right, and the colored paths indicated the citation relationships. Only one primary citation path colored orange was identified, which means the studies published in Molecular/Biology/Genetics journals were mainly cited by the studies published in Molecular/Biology/Immunology journals.

### Countries/Regions and Institutions

**Figure 3:**
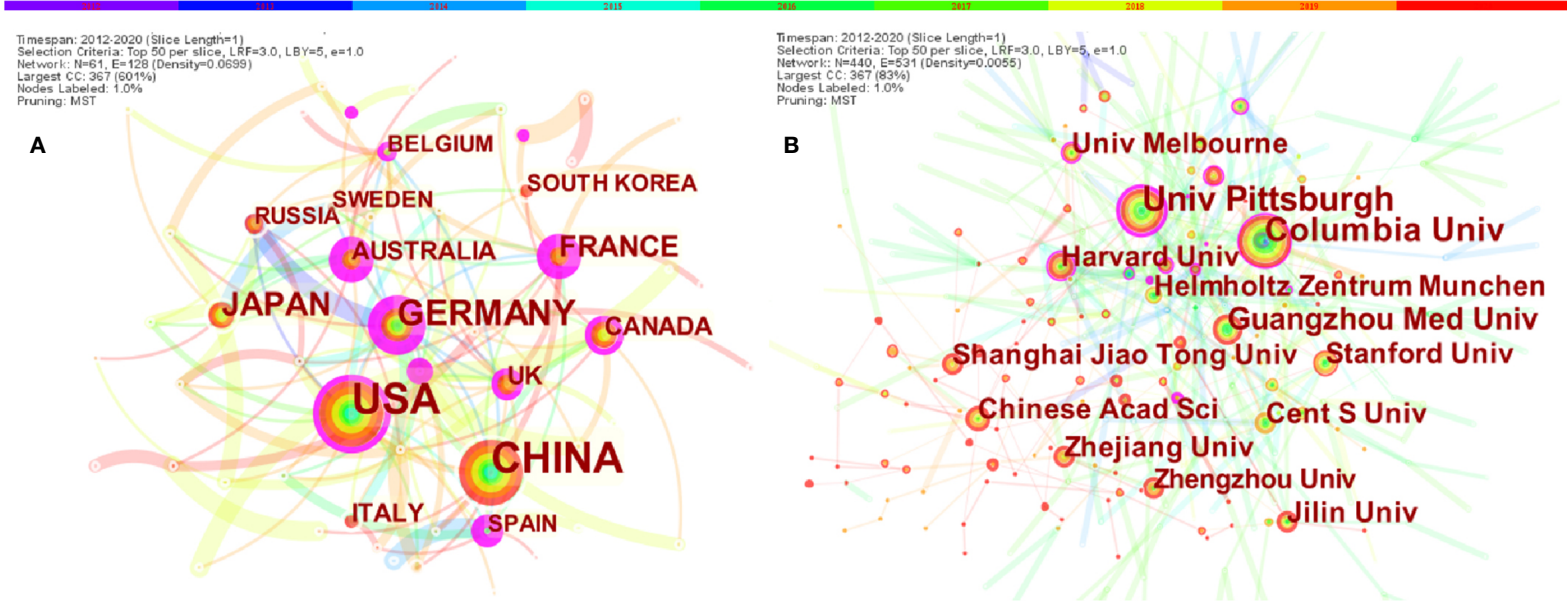
The co-occurrence map of **(A)** countries/regions and **(B)** institutions in ferroptosis research (T≥20). Notes: The size of node reflects the co-occurrence frequencies, and the links indicate the co-occurrence relationships. The color of node and line represents different years, colors vary from purple to red as time goes from 2012 to 2020; and node with purple round means high betweenness centrality (>0.1).

A total of 1,267 publications were co-authored by 438 institutions from 61 countries/regions. The largest number of publications were originated from China (562, 31.03%), followed by the United States (410, 22.64%), Germany (157, 8.67%), and Japan (103, 5.69%) (**Table 3**). Some nodes, such as the United States, France, Germany, Australia, the United Kingdom and Canada, were colored purple round in **Figure 3A** in terms of their high betweenness centrality (≥0.10), which is usually regarded as the important turning points that may lead to transformative discoveries and acts as a bridge (37, 39–41). Furthermore, according to the color of links, the USA (2012), Germany (2013), France (2013), and Russia (2013) were the earliest countries to take up the ferroptosis research. We used minimum spanning tree pruning to make the network clear (**Figure 3A** **)**. Actually, no-pruning countries/regions co-occurrence map contained 61 nodes and 302 links with a density equal to 0.165, indicating active collaborations among different countries/regions. For instance, the USA had cooperation with 34 countries/regions, followed by Germany (n = 30), China (n = 26), France (n = 24), and UK (n = 24).

**Table 3:** The top 10 countries/regions and institutions involved in ferroptosis research.

Rank	Country/region	N (%)	Centrality	Institution	Country/region	N (%)	Centrality
1	China	562 (31.03%)	0.09	University of Pittsburgh	USA	59 (3.34%)	0.20
2	USA	410 (22.64%)	0.18	Columbia University	USA	57 (3.23%)	0.14
3	Germany	157 (8.67%)	0.31	Guangzhou Medical University	China	41 (2.32%)	0.08
4	Japan	103 (5.69%)	0.10	Chinese Academy of Sciences	China	33 (1.87%)	0.08
5	France	55 (3.04%)	0.39	Zhejiang University	China	32 (1.81%)	0.07
6	Australia	49 (2.71%)	0.21	Harvard University	USA	29 (1.64%)	0.11
7	UK	48 (2.65%)	0.19	Stanford University	USA	28 (1.59%)	0.10
8	Canada	42 (2.32%)	0.15	Central South University	China	27 (1.53%)	0.03
9	Italy	39 (2.15%)	0.01	Jilin University	China	25 (1.42%)	0.07
10	Russia	33 (1.82%)	0.02	Helmholtz Zentrum München	Germany	25 (1.42%)	0.06
10				Shanghai Jiao Tong University	China	25 (1.42%)	0.03
10				The University of Melbourne	Australia	25 (1.42%)	0.10

The top 12 institutions were from China (6/12), USA (4/12), Germany (1/12) and Australia (1/12) (**Table 3**). University of Pittsburgh (59, 3.34%) published the most papers, followed by Columbia University (57, 3.23%), Guangzhou Medical University (41, 2.32%), Chinese Academy of Sciences (33, 1.87%), and Zhejiang University (32, 1.81%) (**Table 3**).

### Authors and Co-Cited Authors

**Figure 4:**
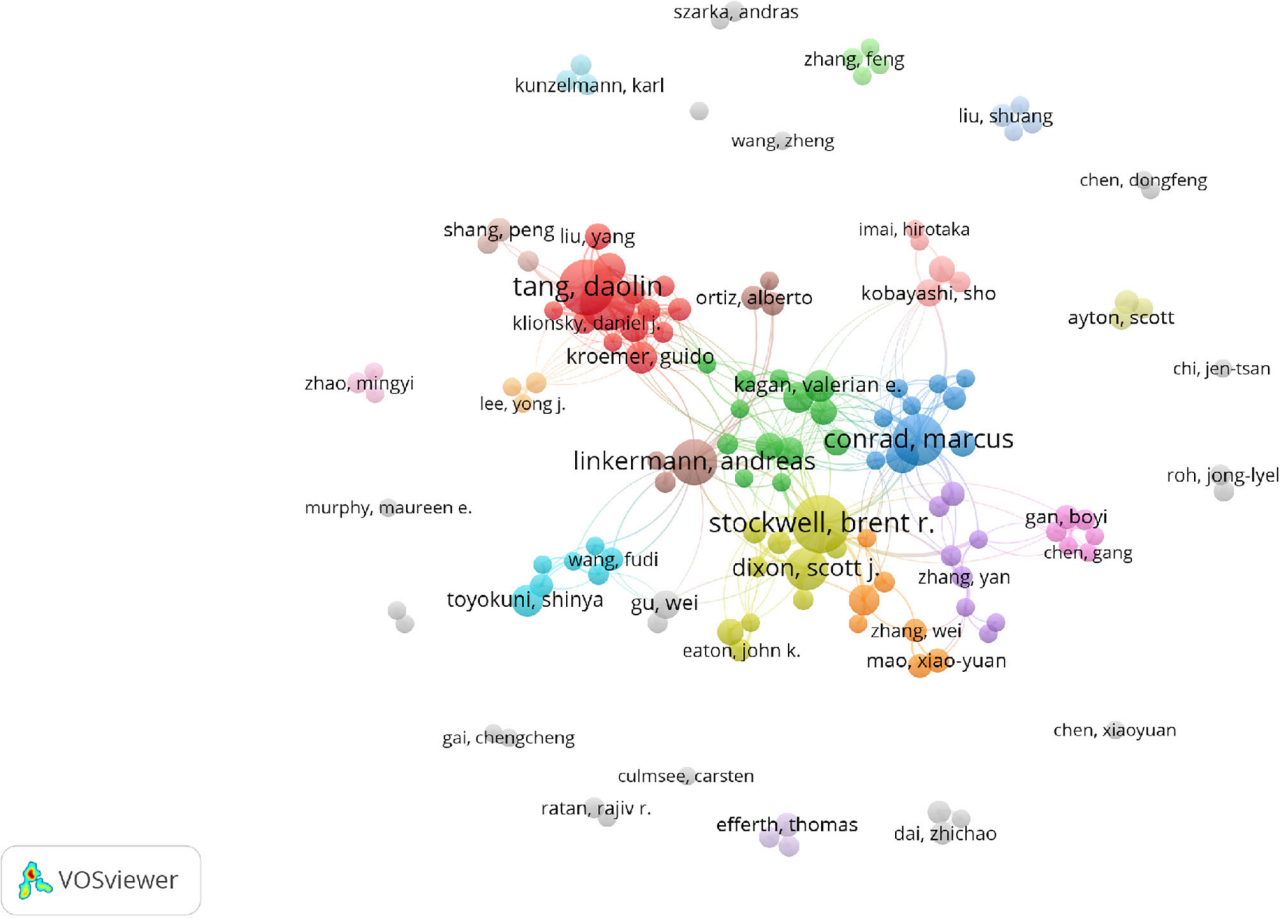
The co-occurrence map of authors in ferroptosis research (T≥5). Notes: The size of node reflects the author’s co-occurrence frequencies, the link indicate the co-occurrence relationship between authors, and the same color of node represent the same cluster.

A total of 6,867 authors were involved in ferroptosis research. Eighteen authors published more than ten articles. Brent R Stockwell published the most papers (n = 39), followed by Daolin Tang (n = 36), Rui Kang (n = 35), Marcus Conrad (n = 31) and Andreas Linkermann (n = 26) (**Table 4**). The authors (n = 135) who published at least five papers (T≥5) were included to build the network map of authors (**Figure 4**). The same color represented the same cluster. There were active collaborations in ferroptosis research, especially among authors in the same cluster, such as Brent R Stockwell and Scott J Dixon, Daolin Tang and Rui Kang, etc. Close cooperation was also observed among clusters, such as Brent R Stockwell and Andreas Linkermann, Brent R Stockwell and Marcus Conrad, Brent R Stockwell, and Xuejun Jiang, etc.

**Table 4:** The top 10 authors and co-cited authors of ferroptosis research.

Rank	Author	Count	Co-cited author	Co-citation
1	Brent R Stockwell	39	Scott J Dixon	1,566
2	Daolin Tang	36	Wan Seok Yang	1,304
3	Rui Kang	35	Jose Pedro Friedmann Angeli	557
4	Marcus Conrad	31	Minghui Gao	519
5	Andreas Linkermann	26	Andreas Linkermann	462
6	Scott J Dixon	22	Brent R Stockwell	462
7	Jose Pedro Friedmann Angeli	14	Sebastian Doll	413
8	Valerian E Kagan	13	Lorenzo Galluzzi	383
9	Guido Kroemer	13	Yangchun Xie	344
10	Jiao Liu	13	Li Jiang	327
10	Shinya Toyokuni	13		

**Figure 5:**
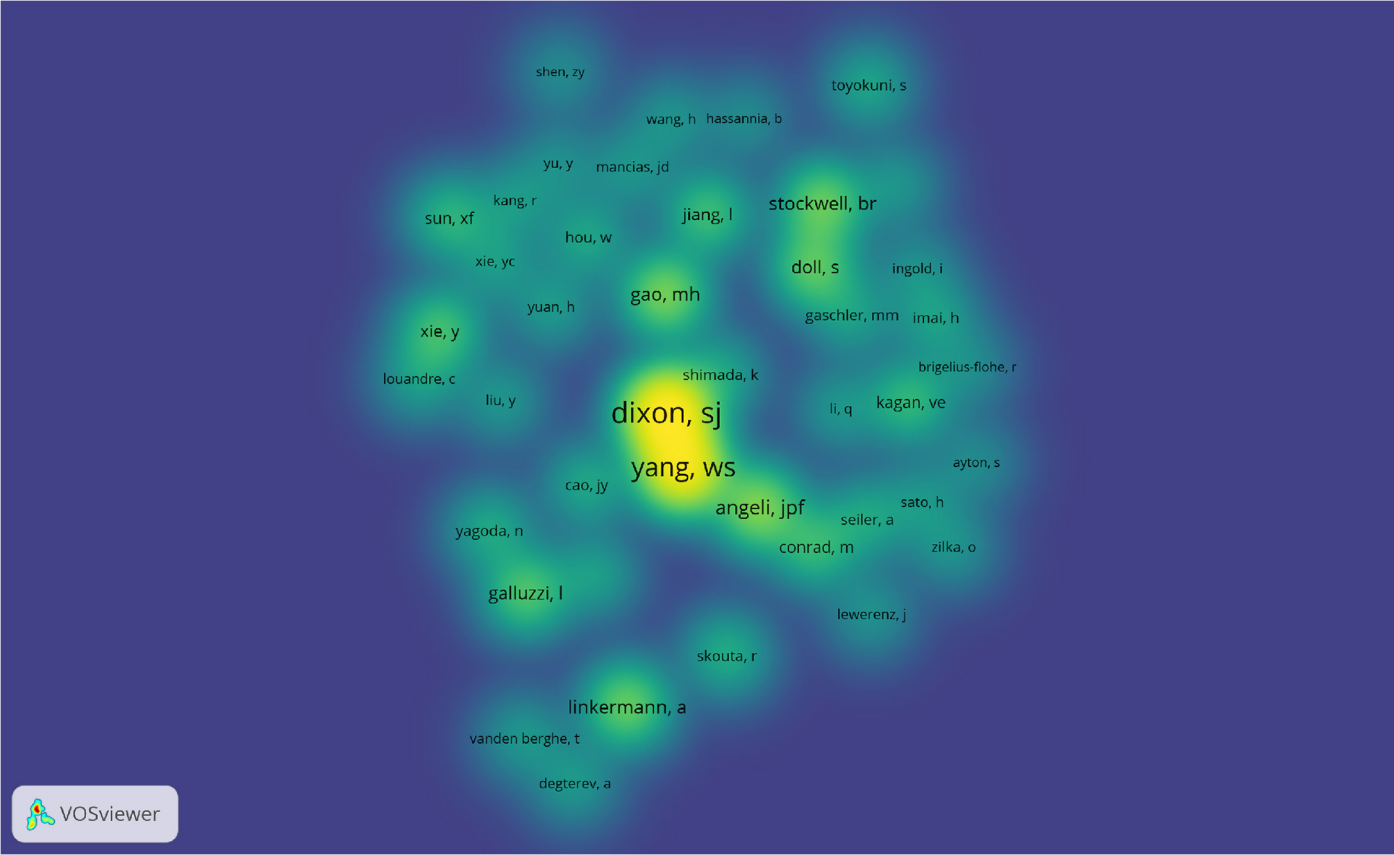
The density map of co-cited authors in ferroptosis research (≥100). Notes: The size of word, the size of round, and the opacity of yellow is positively related to the co-cited frequency.

Co-cited authors are authors who have been co-cited together in a range of publications (42). Among 36,666 co-cited authors, 16 were co-cited over 200. Scott J Dixon (n = 1566) ranked first, followed by Wan Seok Yang (n = 1304), Jose Pedro Friedmann Angeli (n = 557), Minghui Gao (n = 519), Andreas Linkermann (n = 462), and Brent R Stockwell (n = 462). The remaining four top authors were co-cited from 327 to 413 (**Table 4**). The authors (n = 43) with co-citations of at least 100 (T ≥ 100) were used to make the density map (**Figure 5**); this type of knowledge-map could present the high-frequency co-cited authors clearly. According to **Figure 5**, Scott J Dixon and Wan Seok Yang had the hottest color for the most co-cited.

### Keyword Co-Occurrence, Clusters, and Evolution

**Figure 6:**
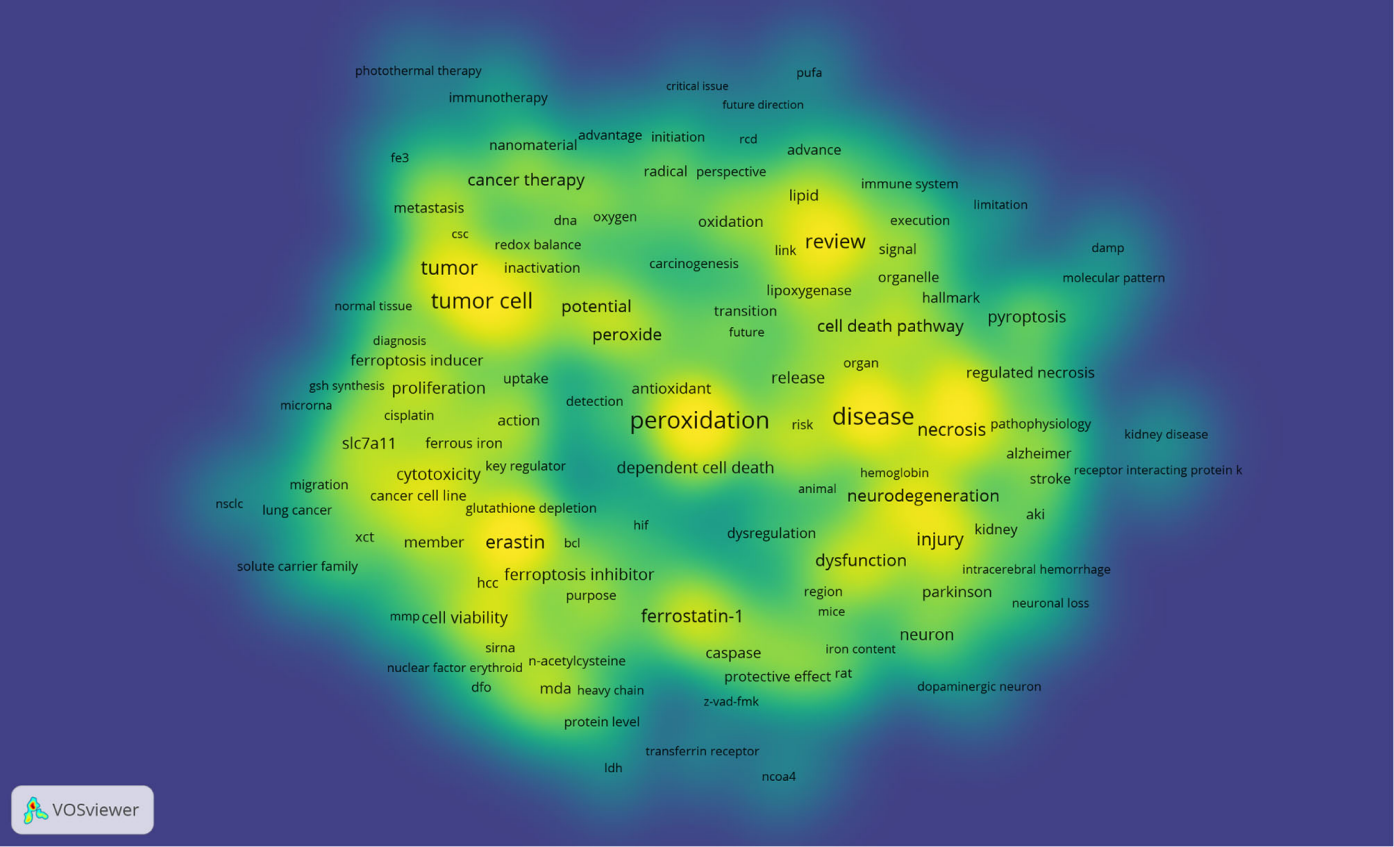
The density map of terms in ferroptosis research (T≥10, relevance score ≥0.081) Notes: The size of word, the size of round, and the opacity of yellow is positively related to the co-occurrence frequency.

**Figure 7:**
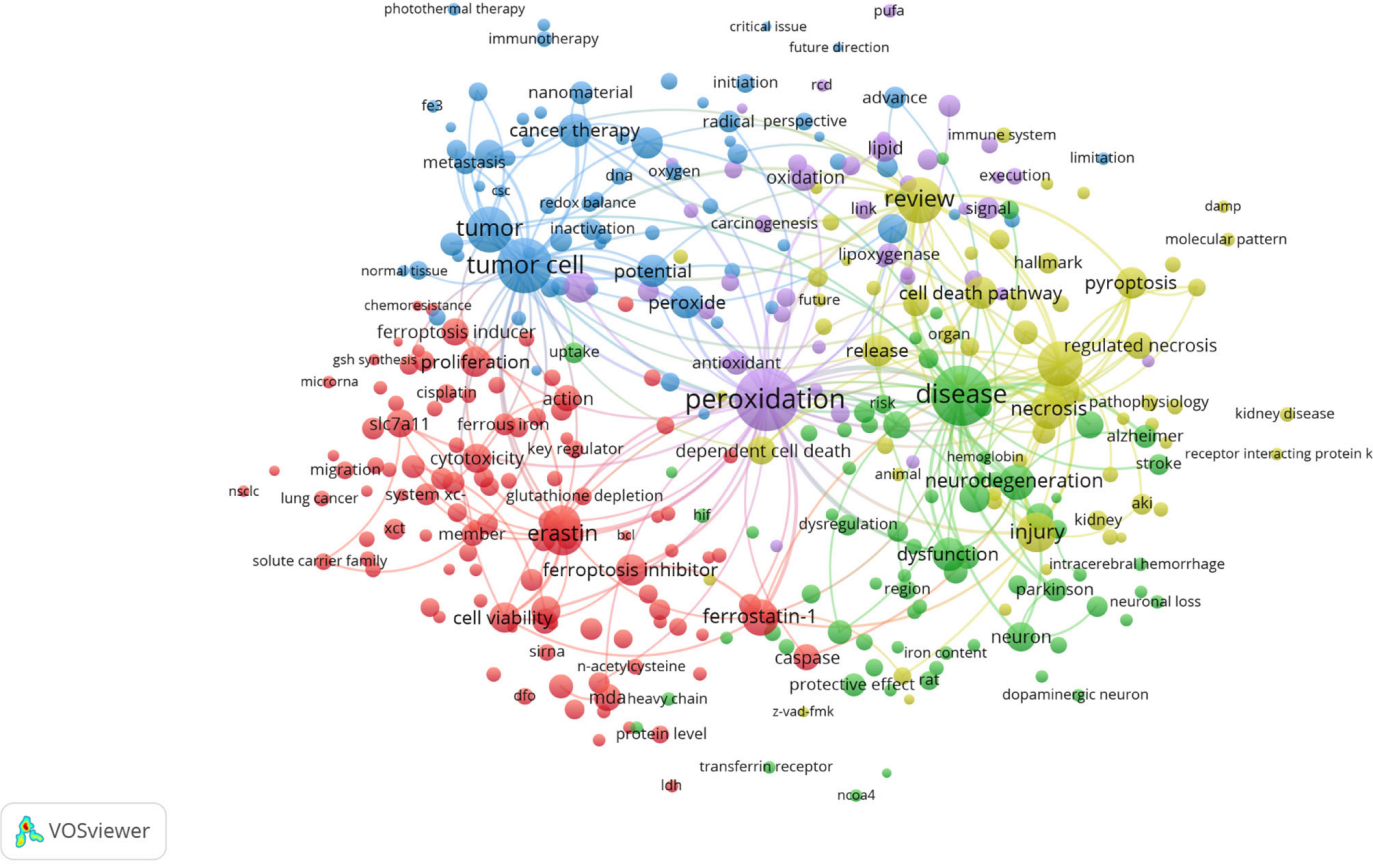
Terms co-occurrence network and clusters in ferroptosis research (T≥10, relevance score ≥0.081, contains 357 items, 5 clusters and 25,151links; max lines = 200.). Notes: The size of node and word reflects the co-occurrence frequencies, the link indicate the co-occurrence relationship, and the same color of node represent the same cluster.

VOSviewer was used to present the term co-occurrence (**Table 5**, **Figure 6**, **7**) and cluster analysis (**Figure 7**). A total of 25,413 terms were extracted, of which 595 appeared more than ten times and 51 appeared more than 100 times. The density map (**Figure 6**) of terms can find the high-frequency co-occurrence terms, which reveal the hotspots in a specific research field. As we can see from **Figure 6** and **Table 5**, peroxidation was the most important term with 350 (2.97%) co-occurrences, followed by disease, tumor cell, tumor, review and necroptosis.

**Table 5:** The top 20 terms of ferroptosis research (relevance score>0.081).

Rank	Term	Count	Rank	Term	Count
1	peroxidation	350 (2.97%)	12	neurodegeneration	106 (0.90%)
2	disease	321 (2.73%)	13	cancer therapy	99 (0.84%)
3	tumor cell	258 (2.19%)	13	inflammation	99 (0.84%)
4	tumor	186 (1.58%)	15	dysfunction	96 (0.82%)
5	review	185 (1.57%)	16	peroxide	92 (0.78%)
6	necroptosis	168 (1.43%)	17	potential	88 (0.75%)
7	erastin	148 (1.26%)	18	cell death pathway	86 (0.73%)
8	injury	141 (1.20%)	19	release	85 (0.72%)
9	necrosis	121 (1.03%)	20	ferroptosis inhibitor	84 (0.71%)
10	cell line	113 (0.96%)	20	pyroptosis	84 (0.71%)
11	ferrostatin-1	111 (0.94%)	20	sensitivity	84 (0.71%)

Cluster analysis can show the knowledge structure of the research field (29). According to the link strength of term co-occurrence, the network was divided into five clusters (**Figure 7**). It is highly homogeneous between the terms in one cluster. Cluster 1 (red) is the largest cluster with 110 co-occurrence terms: erastin, cell line, ferrostatin-1, cell viability, knockdown, cytotoxicity, cell viability, SLC7A11, malonaldehyde (MDA), sorafenib, caspase, ferrous iron, system xc-, RSL3, miRNA, siRNA, etc. The topic of Cluster1 is the mechanism of ferroptosis. Cluster 2 (green) is mainly related to nervous system injury, which includes 73 terms: disease, neurodegeneration, dysfunction, pathogenesis, neuron, iron homeostasis, Parkinson’s disease, Alzheimer, stroke, neuronal death, etc. Cluster 3 (blue) focuses on cancer, which contains 69 terms: tumor cell, tumor, anti-cancer therapy, peroxide, potential, application, chemotherapy, nanomaterial, new strategy, tumor microenvironment, photothermal therapy, photodynamic therapy, etc. Cluster 4 (yellow) is mainly related to cell death with 63 terms: review, necroptosis, necrosis, injury, inflammation, cell death pathway, pyroptosis, regulated necrosis, cell death mechanism, ischemia-reperfusion, acute kidney injury (AKI), etc. Cluster 5 (purple) is related to lipid peroxidation, which includes 42 terms: peroxidation, oxidation, lipid, antioxidant, phospholipid, lipoxygenase, lipid hydroperoxide, acyl-CoA synthetase long chain family member 4 (ACSL4), polyunsaturated fatty acid (PUFAs), etc.

**Figure 8:**
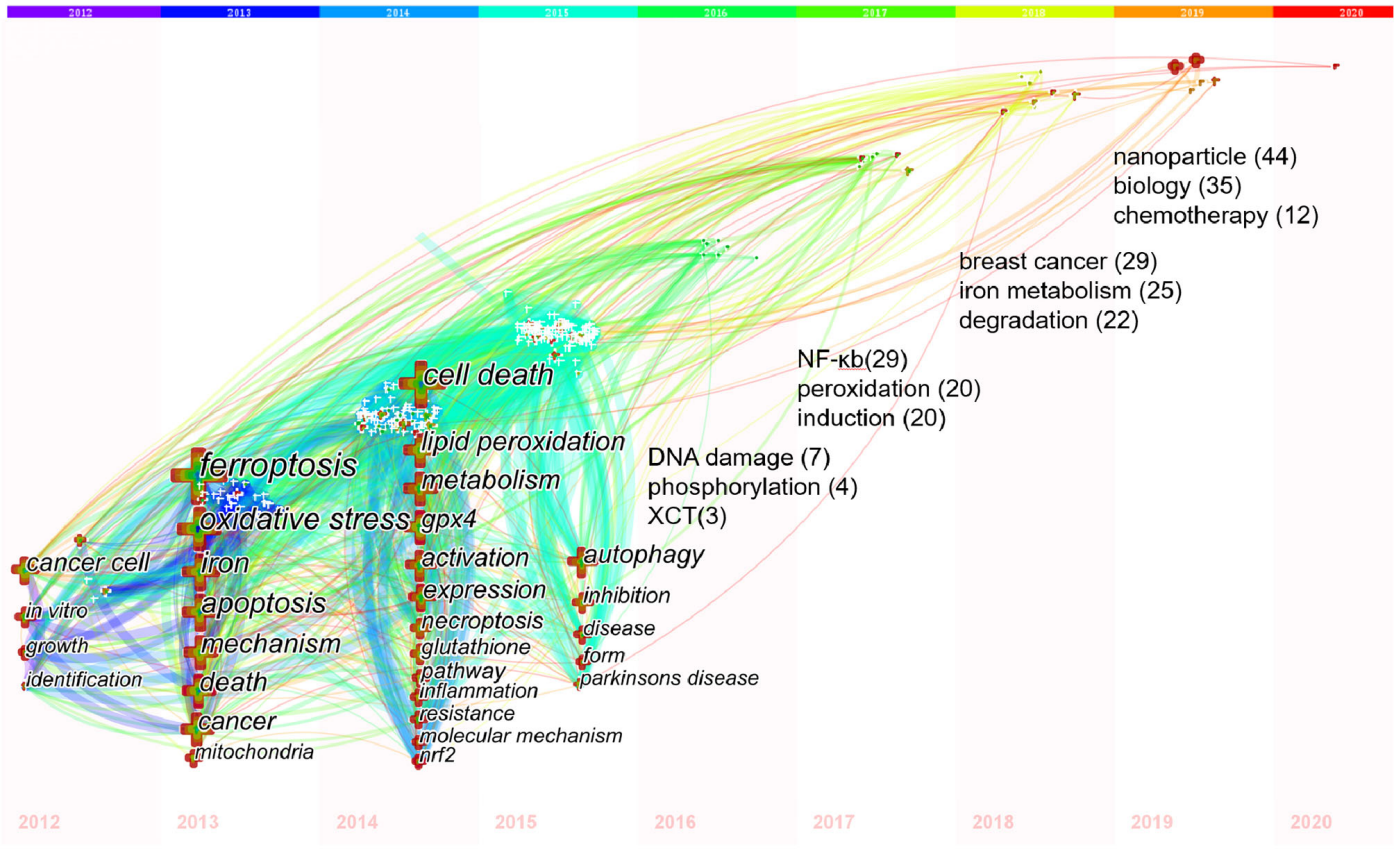
Keywords timezone view of ferroptosis research. Notes: In 2012-2015, keywords with co-occurrence ≥50 were showed; In 2016-2019, the annual top3 keywords were showed with its co-occurrence frequency. The size of cross and word reflects the co-occurrence frequencies, the link indicate the co-occurrence relationship. The color of node and line represents different years, colors vary from purple to red as time goes from 2012 to 2020.

Keywords time zone view was designed by CiteSpace, which could show the evolution of high-frequency keywords clearly. Keywords were located in the year they first co-occurred, and the color of links represents the first year two keywords appear simultaneously. High-frequency keywords (T≥50) were shown in **Figure 8**, while the threshold was a cumulative figure, leading some latest keywords had not accumulated 50 enough. Consequently, we added the annual top three high-frequency keywords from 2016 to 2019 to supplement the timezone map (**Figure 8**). Among them, nuclear factor erythroid-2 related factor 2 (Nrf2) may have turning point significance with a high centrality (0.12) more than 0.10 (41).

### Co-Cited Reference and Reference Burst

We used CiteSpace to detect the co-cited references. **Table 6** showed that the top 10 co-cited references were co-cited at least 196 times, especially three of them were co-cited over 300 times. The most co-cited reference was a review published in *Cell* by Brent R Stockwell, etc. in 2017 (1), entitled “Ferroptosis: A Regulated Cell Death Nexus Linking Metabolism, Redox Biology, and Disease”, followed by an article entitled “Regulation of ferroptotic cancer cell death by GPX4” (3).

**Table 6:** Top 10 co-cited references for ferroptosis research.

Rank	ID	Title	Journal	Co-citation
1	Stockwell BR (1)	Ferroptosis: A Regulated Cell Death Nexus Linking Metabolism, Redox Biology, and Disease	Cell	433
2	Yang WS (3)	Regulation of ferroptotic cancer cell death by GPX4	Cell	374
3	Xie Y (43)	Ferroptosis: process and function	Cell Death Differ	335
4	Jiang L (44)	Ferroptosis as a p53-mediated activity during tumor suppression	Nature	279
5	Doll S (45)	ACSL4 dictates ferroptosis sensitivity by shaping cellular lipid composition	Nat Chem Biol	276
6	Yang WS (46)	Ferroptosis: Death by Lipid Peroxidation	Trends Cell Biol	273
7	Angeli JPF (4)	Inactivation of the ferroptosis regulator Gpx4 triggers acute renal failure in mice	Nat Cell Biol	271
8	Gao MH (47)	Glutaminolysis and Transferrin Regulate Ferroptosis	Mol Cell	240
9	Kagan VE (48)	Oxidized arachidonic and adrenic PEs navigate cells to ferroptosis	Nat Chem Biol	234
10	Yang WS (49)	Peroxidation of polyunsaturated fatty acids by lipoxygenases drives ferroptosis	Proc Natl Acad Sci U S A	196

**Figure 9:**
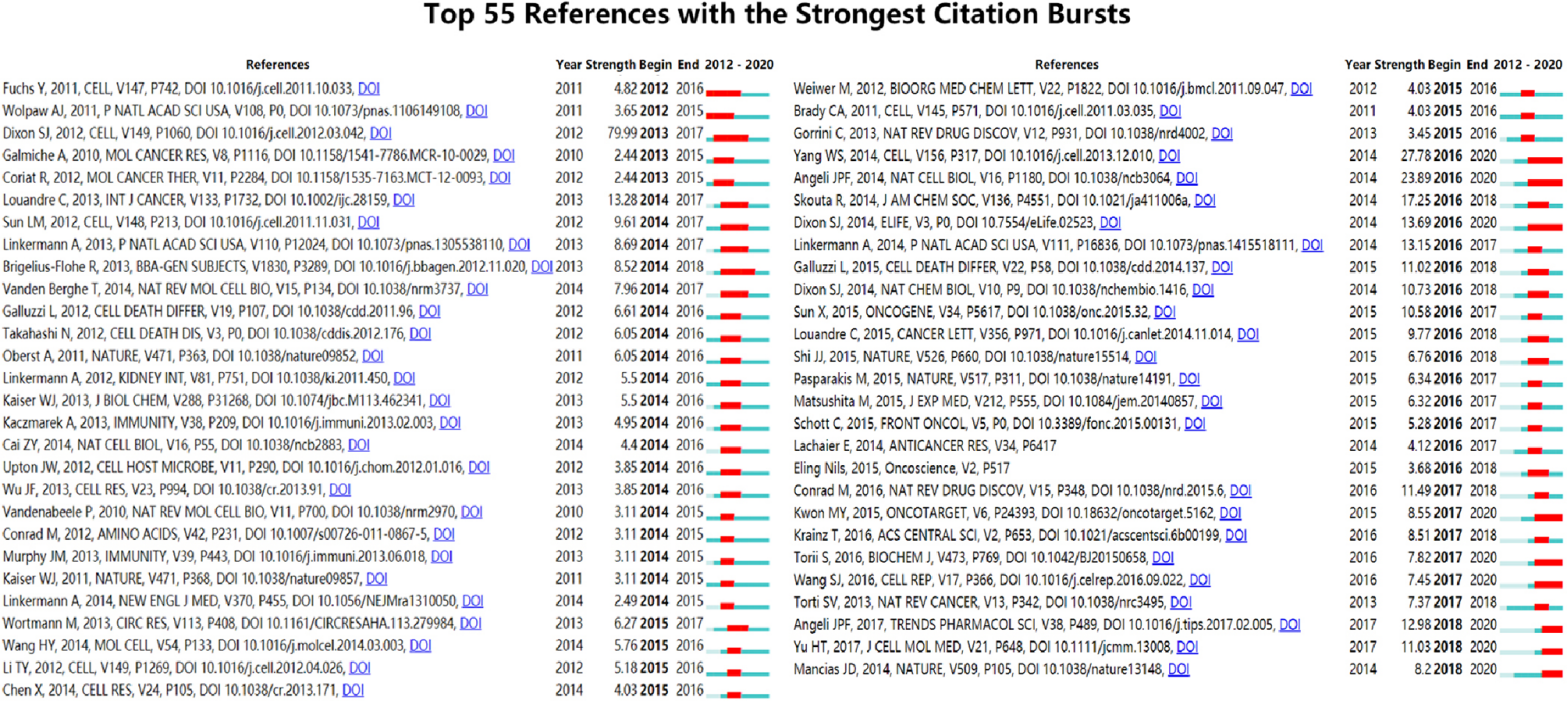
Top 55 references with the strongest citation bursts (sorted by the beginning year of burst). Notes: The Blue bars mean the reference had been published; the red bars mean citation burstness.

References with citation bursts are defined as those that are cited frequently over a while (41). In CiteSpace, we set the burst duration to at least two years, from which we detected 55 references with the strongest citation bursts (**Figure 9**). **Figure 9** showed that 34.55% (19/55) of the references appeared citation burstness in 2014, followed by 2016 (15/55,27.27%) and 2015 (7/55,12.73%). Notably, nine references (16.36%) were in burstness until 2020. The paper with the strongest burstness (strength=79.99) was entitled “Ferroptosis: an iron-dependent form of non-apoptotic cell death” (2), published in *Cell* by Scott J Dixon, etc. in 2012, with citation burstness from 2013 to 2017.

## Discussion

### General Information

Based on the data from WoSCC database up to November 1, 2020, a total of 1,267 ferroptosis researches were published in 466 academic journals by 6,867 authors in 438 institutions from 61 countries/regions.

Change of the annual output is an essential indicator for the development trend in the field (29, 35). The ferroptosis research officially started in 2012, the year Scott J Dixon presented “ferroptosis” (2), and showed an upward tendency overall (**Figure 1**). It could be divided into three stages, namely, “Germination,” “Stable growth,” and “Rapid development.” “Germination”(2012–2013): The concept of ferroptosis was officially proposed (2), and there were four articles in these 2 years. “Stable growth” (2014–2017): In this stage, ferroptosis gained more scientists’ interest and the annual output grew steadily. “Rapid development” (2018 to present): During this period, the number of annual publications was approximately twice that of the previous year, indicating that ferroptosis research has attracted mounting researchers’ attention and developed rapidly. Furthermore, the increasing trend looks promising.

Journals and co-cited journals analysis (**Table 1**) showed that *Cell Death & Disease* published the most ferroptosis research, while *Cell* received the largest number of co-cited references. Both of these are journals on cell biology, which is consistent with the dual-map analysis (**Figure 2**). The dual-map overlay of journals stands for the topic distribution of academic journals (38); **Figure 2** showed only one main citation path from Molecular/Biology/Genetics co-cited journals to Molecular/Biology/Immunology journals, implying that ferroptosis-related studies are focused on basic research nowadays, while researches on translational medicine is still limited (7). Meanwhile, journals at the Q1 JCR division with high IF accounted for the majority of top 10 journals (70%) and co-cited journals (80%), suggesting that these journals have interests and play essential roles in ferroptosis-related researches.

Are there differences among countries/institutions in ferroptosis studies? **Table 3** and **Figure 3** showed that China, the USA, and Germany were the top 3 productive countries. However, the USA, France, Germany, Australia, the United Kingdom, and Canada were regarded as important turning points that may lead to transformative discoveries (37, 39–41). Furthermore, the United States was the earliest country to take up the ferroptosis study, followed by Germany, France, and Russia; these four countries were also the top 10 productive countries. Indicating that the United States is always a productive and influential country in ferroptosis research; noticeably, China started later but has emerged as one of the most productive contributors in recent years. That is consistent with the finding in neuroscience research, and may be related to the economic development and financial input into academic research of these countries (50). Besides, there were active collaborations among different countries/regions, especially the United States, indicating ferroptosis-related research had gained interest worldwide, and the United States was the main collaborating center. The top 12 institutions were from four countries; three-fifths were from China, while the top 2 were from the USA. The University of Pittsburgh, Columbia University, and Guangzhou Medical University published the most. Moreover, we found active cooperation among the University of Pittsburgh, Columbia University, Harvard University, and other institutions, implying their notable contributions to the ferroptosis field.

Highlighting the contributions of influential researchers, such as the authors with many co-occurrences or co-cited papers in a specific field, can help scholars move along the road and provide further directions and guidelines (51). In our analysis (**Table 4**, **Figures 4** and **5**), Brent R Stockwell published the most papers, while Scott J Dixon had the most co-citations. Meanwhile, we found four scholars who were not only the top 10 productive authors but also the top 10 co-cited authors, namely Brent R Stockwell, Andreas Linkermann, Scott J Dixon, and Jose Pedro Friedmann Angeli. Implying that these four authors had an outstanding contribution to ferroptosis field. Furthermore, the map of authors and co-cited authors provides information about potential collaborators and influential research groups (52). In the ferroptosis field, researchers have active cooperation within and between institutions, especially among the influential authors. For example, 27 researchers from 24 institutions presented the most co-cited review entitled “Ferroptosis: A Regulated Cell Death Nexus Linking Metabolism, Redox Biology, and Disease” (1). It is suggesting that these influential teams could be potential collaborators for researchers.

### Knowledge Base

Co-cited references are references that have been cited together by other publications. However, the knowledge base is the collection of co-cited references cited by the corresponding research community (41, 53–55), which is not entirely equivalent to highly cited references. In this bibliometric analysis, the top 10 references co-cited by the included ferroptosis literature (**Table 6** **)** were as follows.

In 2017, *Cell* published the most co-cited study (n=430) co-authored by Brent R Stockwell and 26 other scholars (1) outstanding in ferroptosis research. This review summarized the mechanisms of ferroptosis, highlighted connections with other biological and medical areas, and recommended guidelines for studying ferroptosis. Wan Seok Yang et al. (3) published the second co-cited study in *Cell* in 2014. This study found GPX4 is an essential regulator of ferroptotic cancer cell death; before that, GPX4 had been proved to protect against lipid peroxidation (56) and oxidative stress damage (57). The third co-cited publication was published in 2016 by Y Xie et al. (43) This review summarized the mechanisms, signaling pathways, and measuring methods of ferroptosis and discussed the role of ferroptosis in disease. The fourth co-cited paper was published by Le Jiang et al. (44) in *Nature* in 2015. This study showed that p53 inhibits cystine uptake and sensitizes cells to ferroptosis by repressing the expression of SLC7A11, a vital component of the cystine/glutamate antiporter. In 2017, Sebastian Doll et al. (45) established the essential role of ACSL4 in ferroptosis and published the fifth co-cited study. They used two approaches, genome-wide CRISPR-based genetic screen and microarray analysis of ferroptosis-resistant cell lines, to reveal that ACSL4 dictates ferroptosis sensitivity by shaping cellular lipid composition. The sixth co-cited paper was published by Wan Seok Yang et al. (46) in 2016. This review summarized the discovery of ferroptosis, the molecular mechanisms controlling ferroptosis, and its increasingly appreciated relevance to health and disease. *Nature Cell Biology* published the seventh co-cited experiment research by Jose Pedro Friedmann Angeli et al. in 2014 (4). This study used inducible Gpx4(−/−) mice to elucidate an essential role for the GSH/Gpx4 axis in preventing lipid-oxidation-induced acute renal failure. Furthermore, they found Liproxstatin-1, a spiroquinoxalinamine derivative, is a potent ferroptosis inhibitor in cells, Gpx4(−/−) mice, and a pre-clinical model of ischemia/reperfusion-induced hepatic damage. The eighth co-cited paper was published by Minghui Gao et al. (47) in *Molecular Cell* in 2015. This study detected that the iron-carrier protein transferrin and amino acid glutamine are the inducers of ferroptosis; and glutaminolysis, the cell surface transferrin receptor and the glutamine-fueled intracellular metabolic pathway, plays crucial roles in the ferroptosis process. Furthermore, this study proved that inhibiting glutaminolysis can reduce ischemia/reperfusion-induced heart damage. In 2017,* Nature Chemical Biology* published the ninth co-cited study authored by Valerian E Kagan et al. (48). This study used quantitative redox lipidomics, reverse genetics, bioinformatics, and systems biology to detect the peroxidation mechanism of ferroptosis, and discovered that oxidized arachidonic and adrenic PEs navigate cells to ferroptosis, which may be useful to anti-cancer therapy. In 2016, the tenth co-cited paper was published by Wan Seok Yang et al. (49) in *PNAS*. They demonstrated that PUFAs are susceptible to lipid peroxidation by lipoxygenases and hence execute ferroptosis.

Generally, the top 10 co-cited references focused on reviews (three reviews were published in 2017 and 2016), mechanisms (include targets and genes, such as GPX4, glutamine, GSH, iron-carrier protein transferrin, p53, SLC7A11, ACSL4, system xc−, PUFAs, lipoxygenase, etc.), and related diseases of ferroptosis (such as cancers, acute renal failure, ischemia/reperfusion-induced hepatic and heart damage etc.), all these were the foundations of ferroptosis research.

### Hotspot Evolution, Knowledge Structure, and Emerging Topics

In bibliometrics, keywords/terms co-occurrence (**Table 5** and **Figure 6**) can reflect the hotspots of an academic field (58), and the timezone view (**Figure 8**) can show the evolution of new hotspots (59). The high-frequency terms of ferroptosis (**Table 5** and **Figure 6**) included peroxidation, inflammation, disease, tumor, cancer therapy, neurodegeneration, review, necroptosis, pyroptosis, etc., which were regarded as the hotspots in ferroptosis research. As time goes on, emerging topics occurred continuously (**Figure 8**). In the germination stage (2012–2013), rising terms included cancer cell, oxidative, mechanism, mitochondria, etc. While in the stable-growth stage (2014–2017), new terms contained more mechanism detection and pay more attention to different diseases, including lipid peroxidation, GPX4, glutathione, pathway, inflammation, resistance, molecule mechanism, Nrf2, disease, Parkinson’s disease, DNA damage, nuclear factor kappa-B (NF-κb), peroxidation, induction, etc. Notably, Nrf2 may have turning significance with a high centrality (41, 60). In the rapid development stage (2018-now), emerging topics, such as breast cancer, iron metabolism, degradation, nanoparticle, biology, did not only continue the characteristics of the stable-growth stage but also used more technology, such as the emerging application of nanoparticles in cancer treatment (61–67). Unfortunately, although many essential regulatory molecules were demonstrated and transferrin receptor 1 protein (TfR1) was considered as a specific marker of ferroptosis by some scholars (68), there are still no acknowledged specific biomarkers of ferroptosis, such as caspase activation for apoptosis or autophagy lysosome formation for autophagy (9, 69).

Moreover, the cluster of keywords/terms could describe the internal knowledge structure and reveal the research frontier of the discipline (29). Cluster analysis showed five main clusters in the ferroptosis field (**Figure 7**), including regulation mechanisms, nervous system injury (including neurodegenerative disease), cancer, the relationship with other types of cell death, and lipid peroxidation, representing five main aspects of ferroptosis research to some extent. As we know, there is cross-talk between ferroptosis and other types of cell death in some similar signals and molecular regulators [e.g., apoptosis (70) and autophagy (71)], while the mechanisms that direct cells to choose among different cell death ways are still an enigma (7). Besides, lipid peroxidation is proved to be a vital mechanism in ferroptosis, but it is still unknown why and how lipid peroxidation leads to the death of cells in ferroptosis, and this is regarded as one of three key areas of future ferroptosis research (72). As for disease, ferroptosis in cancers has been a hotspot from initial stage to now, and there is also a large amount of research on nervous system injury.

References with strong citation bursts (**Figure 9**) could also characterize the emerging topics of a field (35, 41, 73). The strongest citation burstness came from a landmark study published by Dixon SJ et al. (2) in 2012 (79.30, 2013–2017), which coined the term ferroptosis. Early research only recognized that cysteine is necessary to maintain the biosynthesis of glutathione and inhibit a type of cell death in mammalian cells (74, 75) that is also preventable by iron chelators or lipophilic antioxidants (76). While in this study, Dixon SJ’s team found that erastin could trigger a unique iron-dependent form of non-apoptotic cell death and named it ferroptosis. Since then, additional compounds and regulatory mechanisms have been identified, and ferroptosis becomes an emerging focus of regulated cell death. More importantly, among the top 55 references with the strongest citation burst (**Figure 9**), nine references are still in burstness. These nine references represent the latest emerging topics of ferroptosis hence they deserve further discussion (19, 35). Ranking by burstness strength, the first paper (strength=27.78) was published by Wan Seok Yang et al. (3) in *Cell* in 2014, with the citation burstness lasted for five years (2016–2020), proving that GPX4 is an essential regulator of ferroptotic cancer cell death. Jose Pedro Friedmann Angeli et al. (4) detected that inactivation of Gpx4 triggers acute renal failure in mice. The study was published in *Nature Cell Biology* in 2014 with the second strongest citation burstness (strength=23.89) lasting for five years (2016–2020). Scott J Dixon et al. (77) published the third reference in 2014 (13.69, 2016–2020). They found that erastin is a potent inhibitor of system xc− function, which is much more potent than sulfasalazine, the known best inhibitor of system xc−. They also discovered that the anti-cancer drug sorafenib inhibits system xc− function and could trigger endoplasmic reticulum stress and ferroptosis. The reference with the fourth-strongest citation burstness was published in *Trends in Pharmacological Science* by Jose Pedro Friedmann Angeli et al. (78) in 2017 (12.98, 2018–2020). This review mainly summarized the fundamental aspects of lipid peroxidation in ferroptosis and their potential contribution to disease; furthermore, they discussed the potential pharmacological approaches aiming to subvert lipid peroxidation and suppress ferroptosis. The fifth paper is a review authored by Haitao Yu et al. (79) in 2017 (11.03, 2018–2020), focusing on the relationship between ferroptosis and human tumorous diseases. Min-Young Kwon et al. (80) published the sixth study in 2015 (8.55, 2017–2020). They elucidated that heme oxygenase-1 (HO-1) accelerates erastin-induced lipid peroxidation and ferroptosis. The seventh reference (81) was published in *Nature* in 2014 (8.2, 2018–2020). This work identified nuclear receptor coactivator 4 (NCOA4) as a selective cargo receptor for autophagic turnover of ferritin. In previous studies, genetic overexpression or knockdown of NCOA4 has been shown to trigger or prevent erastin-induced ferroptosis in several (82). Indeed, ferroptosis has been suggested as a type of autophagy-dependent cell death (71). The eighth study (83) (7.82, 2017–2020) detected that functional lysosomes play an essential role in functional lysosomes in the ferroptosis of cancer cells. Moreover, the ninth study (84) (7.45, 2017-2020) demonstrated the vital role of p53 acetylation in ferroptosis and its remaining tumor suppression activity. The citation burstness analysis showed that exploring the mechanism of ferroptosis (such as GPX4, lipid peroxidation, HO-1, NCOA4, functional lysosomes, p53 acetylation, etc.) and applying to related disease (such as tumor, acute renal failure, etc.) were the recent major topics in the field of ferroptosis research.

From the above analysis, we can see that ferroptosis research initially focused on experimental research and cancer. Afterward, in-depth experimental research detected more star mechanisms (such as GPX4, Nrf2, GSH, p53, SLC7A11, ACSL4, system xc−, phospholipids, NAD(P)H, CoQ10, lipid peroxidation, etc.), used new technologies, and related ferroptosis to more diseases (such as kinds of cancer, Alzheimer, Parkinson’s disease, stroke, ischemia-reperfusion damage, kidney disease, liver disease, atherosclerosis, drug resistance, etc.). Furthermore, ferroptosis research began to do clinical trials (85), while there are still no human intervention trials, indicating that the clinical translational application of ferroptosis theories is still ongoing (12). The reason might be that the ferroptosis inhibitors such as ferrostatin-1 and liproxstatin-1 may inhibit other ROS-dependent forms of cell death, although they are safe in pre-clinical animal studies (7). In the latest traditional review, Daolin Tang (7) pointed out that we are at the dawn of ferroptosis research; challenges require more specific drugs, sophisticated pre-clinical models, and innovative technology. That is consistent with our bibliometric detecting.

### Limitations

This study also comes with certain limitations inherent in bibliometrics. Firstly, data were retrieved only from the WoSCC database, while a few studies not included in WoSCC were missed. However, WoSCC is the most commonly applied database for scientometric analysis (19, 28); data from WoSCC could represent most information in a degree. Secondly, all information was extracted by bibliometric tools basing on machine learning and natural language processing, which may lead to bias as reported in other bibliometric studies (86). Nevertheless, compared to the latest traditional reviews (7, 9, 72, 87, 88), our results are basically consistent with them while providing researchers with richer objective information, knowledge and insight.

## Conclusion

In conclusion, ferroptosis research is in a rapid development stage with active cooperation worldwide, of which the United States is the main collaborating center. Current publications are mainly in the molecular and biology field. Five main aspects of ferroptosis research included regulation mechanisms, nervous system injury, cancer, relationships with other types of cell death, and lipid peroxidation. The latest hotspots are nanoparticle, cancer therapy, iron metabolism, and in-depth mechanism. Notably, Nrf2 may have turning significance. Based on the results, the emerging topics would be the further regulatory mechanism of ferroptosis and the broader application of ferroptosis-related disease with advanced technology.

Overall, this is the first study to systematically analyze the ferroptosis-related publications by bibliometric and knowledge-map. Moreover, we analyzed data by both CiteSpace and VosViewer, which could obtain richer results from different perspectives. Compared to traditional reviews, this study provides an original and objective insight into ferroptosis research. We believe the results of this study would provide helpful references for further research.

## Author’s Note

The data used for analysis is in Annexes 1, and the result data is in Annexes 2.

## Data Availability Statement

The original contributions presented in the study are included in the article/**Supplementary Material**. Further inquiries can be directed to the corresponding authors.

## Author Contributions

HX and JJ designed this study. YF collected the data. JZ and LX performed the analysis. TW and WT normalized the pictures. JZ and LS wrote the original draft. All authors contributed to the article and approved the submitted version.

## Funding

The work was supported by the National Natural Science Foundation of China (No. 81874412), National Natural Science Foundation of China (No.82004145), Beijing Natural Science Foundation (No.7204298), and Central Public Welfare Research Institutes of China Academy of Chinese Medical Sciences (No. ZZ13-YQ-017).

## Conflict of Interest

The authors declare that the research was conducted in the absence of any commercial or financial relationships that could be construed as a potential conflict of interest.
